# Saponin-Mediated Isolation of Megacomplexes of the Mitochondrial Oxidative Phosphorylation System

**DOI:** 10.3390/biology15201826

**Published:** 2026-10-13

**Authors:** Cunyu Peng, Peixia Hu, Guolin Li, Meichi Wang, Zhigui Duan

**Affiliations:** 1 Center for Aging Biomedicine, The National & Local Joint Engineering Laboratory of Animal Peptide Drug Development, College of Life Sciences, Hunan Normal University, Changsha 410081, China; pengcunyu0607@163.com (C.P.); 18339443703@163.com (P.H.); hnsdlgl@hunnu.edu.cn (G.L.); 13464@hunnu.edu.cn (M.W.); 2 National Demonstration Center for Experimental Biology Education, Hunan Normal University, Changsha 410081, China; 3 Hunan Research Center of the Basic Discipline Developmental Biology, College of Life Sciences, Hunan Normal University, Changsha 410081, China

**Keywords:** *Sapindus saponaria* saponins, mass spectrometry, oxidative phosphorylation supercomplex, size exclusion chromatography, co-immunoprecipitation

## Abstract

The assembly of mitochondrial oxidative phosphorylation (OXPHOS) supercomplexes determines electron flux and ATP synthesis. Although several techniques have been developed to isolate these supercomplexes, their native assembly states within the inner mitochondrial membrane have remained unclear. Here, we developed a separation technique based on saponins from *Sapindus saponaria* pericarps that enables the isolation of megacomplexes from mitochondria. Using size-exclusion chromatography (SEC) coupled with Western blot, we found that these megacomplexes may represent the predominant assembly state of the mitochondrial OXPHOS system. Furthermore, co-immunoprecipitation followed by Western blot assays revealed a megacomplex comprising respiratory chain complexes I–IV and ATP synthase. Notably, these supercomplexes remained intact under both fasting and a 24-h high-fat diet, underscoring their stability in vitro. Collectively, this study defines the primary architectural organization of OXPHOS system and establishes the saponin-based method as a mild alternative for extracting membrane protein supercomplexes.

## Simple Summary

Current in vitro isolation methods for the mitochondrial oxidative phosphorylation system yield only supercomplexes and individual free complexes, leaving the native assembly of these complexes uncertain. The present study aimed to develop a novel reagent for the efficient extraction of mitochondrial oxidative phosphorylation megacomplexes, thereby enabling elucidation of their physiological assembly state. Using a native saponin reagent, we extracted numerous oxidative phosphorylation megacomplexes comprising respiratory complexes and ATP synthases. Subsequent analysis concluded that these megacomplexes constitute the dominant functional units of the mitochondrial oxidative phosphorylation system. Furthermore, using these optimized procedures, we successfully isolated and characterized a higher-order oxidative phosphorylation megacomplex comprising all five oxidative phosphorylation complexes from rat liver mitochondria. Collectively, these findings suggest that oxidative phosphorylation megacomplexes may represent the primary architectural organization of the oxidative phosphorylation system, and they highlight the potential of *Sapindus saponaria* saponins (SASS) as a promising reagent for isolating and purifying large membrane protein supercomplexes in vitro.

## 1. Introduction

The oxidative phosphorylation (OXPHOS) system, situated in the inner mitochondrial membrane, is responsible for the majority of ATP synthesis in eukaryotic cells. This process is mediated by respiratory complexes I–IV (CI–CIV) and ATP synthase (CV), along with the membrane-bound ubiquinone and soluble cytochrome c [1,2,3,4,5,6]. Although the structure of this system has been studied for over half a century, the precise assembly of its complexes remains debated. In the early 1960s, electron micrographs of mitochondrial cristae provided initial support for the solid-state model of the electron transport chain. This model posited that individual respiratory complexes associate to form distinct supramolecular assemblies, termed oxysomes [7]. However, evidence of a diffusion-coupled electron transport mechanism [8] and the successful purification of individual functional respiratory complexes [9,10] subsequently undermined this model, leading to the proposal of the fluid model, wherein all respiratory complexes moved randomly within the inner mitochondrial membrane. Given the growing evidence for diverse respiratory supercomplexes comprising CI, CIII, and CIV [2,3,5,10,11,12,13,14,15,16], the currently accepted random diffusion model (i.e., plasticity model) was proposed to explain the assembly states of the respiratory chain. In this model, respirasomes containing CI, CIII, and CIV in various stoichiometries, other supercomplexes (CICIII or CIII_2_CIV), and free populations of respiratory complex coexist [5,15,17,18,19]. Available data suggest that most CII and ATP synthases remain as free complexes [2,5,17]. Although recent evidence of the interactions between ATP synthase and respiratory complexes has challenged the plasticity model [20,21,22], the isolation of intact OXPHOS supercomplex remains a significant challenge.

The efficient separation and identification of respiratory chain complexes and ATP synthases typically rely on detergents such as dodecylmaltoside (DDM), Triton X-100 (TX) and digitonin (DIG) [17,23,24,25,26,27]. For example, DDM and TX enable the harvesting of free respiratory complexes and ATP synthases [17,23,28]. Although DIG facilitates the isolation of various large respiratory supercomplexes, including Q-respirasomes (CIII_2_–CIV_1–2_) [14], CI–CIV_1–2_, N-respirasomes (CI–CIII_2_–CIV_1–2_) [29], CI_1_–CIII_2_–CIV_1_ [3,18,30] and CI–CIII_2_ [2], a substantial proportion of unassembled free complexes remains detectable in DIG-solubilized mitochondrial lysates. Consequently, it remains unclear whether supramolecular assemblies (CI–CV) truly represent predominant assembly state of the mitochondrial OXPHOS system, or whether current detergent-based separation techniques simply fail to preserve and isolate them. Previous studies have indicated that the dense packing of OXPHOS complexes enhances the kinetic coupling between proton pumping and ATP synthesis [31], and the spatial proximity of respiratory complexes and ATP synthases has been directly visualized on the surface of mitochondrial cristae [22]. These findings suggest that high-order OXPHOS supercomplexes comprising five complexes (CI–CV) likely exist in vivo, despite current analytical capability detect only respiratory supercomplexes, free respiratory complexes, and ATP synthase. This discrepancy is probably attributable to the disruption of higher-order supercomplexes during mitochondrial isolation using the aforementioned detergents [17,32,33]. Hence, to fully elucidate the assembly mechanisms of mitochondrial supercomplexes, novel methodologies capable of isolating intact OXPHOS supercomplexes are urgently needed.

In this study, we incorporated natural *Sapindus saponaria* saponins (SASS)—a mixture of triterpenoid saponins derived from *S. Saponaria* pericarps [34]—into imidazole or Tris-HCl buffers to formulate a novel mitochondrial lysis solution. Mitochondria were gently solubilized with these buffers, and the samples were resolved by blue native polyacrylamide gel electrophoresis (BN-PAGE) or size-exclusion chromatography (SEC). Numerous mega-supercomplexes were thereby isolated from the mitochondrial lysates. Western blotting confirmed that these supercomplexes were OXPHOS megacomplexes, comprising respiratory complexes and ATP synthases, and retained structural integrity throughout solubilization and separation. Quantitative assays suggested that these megacomplexes comprise almost all OXPHOS complexes within mitochondria, thereby corroborating their existence in vitro via our established isolation approach. Furthermore, using these optimized procedures, we successfully isolated and characterized a higher-order OXPHOS supercomplex–termed the “oxphosome”–comprising all five oxidative phosphorylation complexes (CI–CV) from rat liver mitochondria. Collectively, these findings implied that OXPHOS megacomplexes may represent the primary architectural organization of the OXPHOS system, and they highlight the potential of SASS as a promising reagent for isolating and purifying large membrane protein supercomplexes in vitro.

## 2. Materials and Methods

### 2.1. Materials

The SASS (CAS No. 223748-41-2; total saponins ≥95%) were purified from *Sapindus saponaria* pericarp in the Duan laboratory. Briefly, dried *Sapindus saponaria* pericarp powder was extracted with 50% (*v*/*v*) acetic acid–methanol at 50 °C for 30 min. The extract was concentrated under reduced pressure at 60 °C to yield crude saponins. The crude saponins were dissolved in deionized water, and the pH was adjusted to approximately 9.0 with NaOH. After centrifugation of the crude saponin solution at 12,000 g for 10 min, the supernatants were sequentially passed through a D285 macroporous resin column followed by an AB-8 macroporous resin column. The saponins were eluted from the AB-8 column with 70% ethanol, then dried and stored at room temperature.

DDM, DIG (≥50%), and TX were purchased from Aladdin Co., Ltd. (Shanghai, China). Anti-complex I and anti-complex IV immunocapture antibodies were purchased from Abcam (Cambridge, UK). Anti-NDUFS2, anti-SDHA, anti-UQCRC2, anti-MT-CO2, anti-ATP5F1A, anti-CYCS antibodies, and HRP-conjugated goat anti-mouse IgG were purchased from Boster Biotechnology (Wuhan, China). BeyoECL Plus was purchased from Beyotime Biotechnology (Shanghai, China). The resin Seplife 6FF (separation range: 10–4000 kDa) was purchased from Sunresin New Materials (Xi’an, China). Tribromoethanol was purchased from Macklin Biochemical Co., Ltd. (Shanghai, China).

### 2.2. Animal Housing and Ethics

Sprague–Dawley rats (approximately 150 g) were purchased from Hunan SJA Laboratory Animal Co., Ltd. (Changsha, China). All rats were housed under standard specific pathogen-free (SPF) laboratory conditions: controlled ambient temperature at 22 ± 2 °C, relative humidity maintained at 55 ± 10%, and a strict 12-h light/12-h dark cycle with 150–300 lux cage-level illumination. Animals had ad libitum access to autoclaved standard rodent chow and sterile drinking water. After 7 days of acclimatization before formal experiments, all rats were kept in individually ventilated cages with <14 ppm ammonia concentration, following the 3R principles of animal welfare. All rats were deeply anesthetized via intraperitoneal injection of tribromoethanol (300 mg/kg) prior to euthanasia.

### 2.3. Isolation of Mitochondria

Mitochondria were isolated according to the method described by Liao et al. [35], with all procedures performed at 4 °C. Livers were rapidly dissected from the 30-day-old Sprague–Dawley rats (regardless of gender) and rinsed in a physiologic saline. Approximately 2 g of fresh liver, heart, or muscle tissue were minced into fine pieces and then homogenized 8–10 times in a 50-mL glass homogenizer with 20 mL of mitochondrial extraction buffer (250 mmol/L sucrose, 5 mmol/L Tris, 0.5 mmol/L EDTA, pH 7.4) to form a uniform suspension. After centrifuging the homogenate for 10 min at 800 *g*, the supernatants were combined and centrifuged at 10,000 *g* for 10 min. The collected pellets were resuspended in extraction solution and distributed into 8 tubes (approximately 20 mg/tube), and centrifuged again at 10,000 *g* for 10 min. The supernatant was discarded, and the pellets were stored at −20 °C for up to 2 weeks before use.

### 2.4. Preparation of Mitochondrial Protein Samples for BN-PAGE

To analyze mitochondrial supercomplexes by BN-PAGE, extraction was performed as described in Witting et al. [23]. Approximately 20 mg mitochondria (wet weight, containing approximately 2.7 mg of protein) were resuspended in 400 μL of ice-cold sample solubilization buffer (NaCl 50 mM, Imidazole 50 mM, 6-aminohexanoic acid 2 mM, EDTA 1 mM, pH 7.0) and then gently mixed with the indicated amount of 20% DDM (*w*/*v*), 20% TX (*w*/*v*), 20% DIG (*w*/*v*), or 20% SASS (*w*/*v*) by vortexing. The mixtures were incubated for 10 min at room temperature. After centrifuging at 14,000 rpm at 10 °C for 10 min, the collected supernatants were mitochondrial lysate. The detergent/protein (mg/mg) ratio is approximately 18.4, 17.8, 8.1, and 7.3 for SASS, DIG, DDM, and TX.

Protein concentration of these samples was determined using the Lowry method [36], and BSA was used as the standard protein. Absorbance was read at 620 nm using Multiskan FC (Thermo Fisher Scientific, Waltham, MA, USA) after the chromogenic reaction had finished.

### 2.5. BN-PAGE

BN-PAGE was performed according to the method described by Witting et al. [23]. A linear 3.5–16% gradient gel was prepared in a JUNYI mini protein 1.5 mm system using a peristaltic pump. Approximately 100 μg of protein were loaded to each sample well for BN-PAGE.

### 2.6. Immunoblotting of Respiratory Complex CI and CIV

After BN-PAGE, the gel was then electroblotted onto a polyvinylidene fluoride (PVDF) membrane at 100 V for 60 min using a Junyi wet electroblotting apparatus (Junyi, Beijing, China). Immunoblotting was separately performed with anti-complex I or anti-complex IV immunocaptured antibodies. Respiratory CI and CIV signals were detected using an enhanced chemiluminescence (ECL) kit (Beyotime, Shanghai, China) with horseradish peroxidase (HRP)-conjugated goat anti-mouse IgG. The chemiluminescent membrane was imaged using a Tanon-5200 imager (Tanon, Shanghai, China) with an exposure time of 2–5 s.

### 2.7. In-Gel Digestion

Protein bands were excised and placed into tubes. Excised slices were first destained twice with 200 μL of 50 mM NH_4_HCO_3_ in 50% acetonitrile and then dried twice with 200 μL of acetonitrile. The dried pieces of gels were incubated in an ice-cold digestion solution (12.5 ng/μL trypsin in 20 mM NH_4_HCO_3_) for 20 min and then transferred into a 37 °C incubator for overnight digestion. Finally, the tryptic peptides were extracted and lyophilized in a vacuum centrifuge.

### 2.8. Nano-HPLC-MS/MS Analysis

Tryptic peptides were dissolved in 0.1% formic acid (solvent A) and directly loaded onto a reversed-phase analytical column (Acclaim PepMap C18, 75 μm × 25 cm) (Thermo Fisher Scientific, Waltham, MA, USA). The gradient was consisted of an increase from 2% to 30% solvent B (0.1% formic acid in 98% acetonitrile) over 50 min, from 30% to 50% over 5 min, and from 50% to 80% over 1 min followed by holding at 80% for the final 4 min, all at a constant flow rate of 200 nL/min on an EASY-nLC 1200 UPLC system (Thermo Fisher Scientific, Waltham, MA, USA).

The peptides were subjected to nano-electrospray ionization (NSI) followed by tandem mass spectrometry (MS/MS) on Orbitrap Exploris 480 MS (Thermo Fisher Scientific, Waltham, MA, USA) coupled online to the UPLC. The spray voltage was set to 2.3 kV, the funnel RF level to 50, and the heated capillary temperature at 320 °C. For data-dependent acquisition (DDA) experiments, the full MS resolution was set to 60,000 at *m*/*z* 200 and the full MS AGC target was 300% with an injection time (IT) of 25 ms. The mass range was set to 350–1600 *m*/*z*. The AGC target value for fragment spectra was set to 200% with a resolution of 15,000 and an injection time of 50 ms and a Top12 selection mode. The intensity threshold was kept at 2 × 105. The isolation width was set to 1.6 *m*/*z*. The normalized collision energy was set to 30%.

### 2.9. Database Searching, Subcellular Location, Molecular Function Analysis, and Protein–Protein Interaction Prediction

Tandem mass spectra were extracted using Proteome Discoverer software (Thermo Fisher Scientific, version 2.4). The tandem mass spectra were searched against the SwissProt rat database assuming trypsin as the digestion enzyme. The mass error was set to 10 ppm for precursor ions and 0.02 Da for fragment ions. Carbamidomethylation of cysteine was specified as fixed modification in Mascot. Oxidation of methionine was specified in Mascot as a variable modification. Peptide confidence was set to high, and peptide ion score threshold was set at >20.

Proteins identified with Sequest HT Score > 99 were submitted to UniProt (https://www.uniprot.org/) to obtain subcellular location and function information.

The accession numbers of these highly abundant proteins identified from the mega-supercomplex band of the SASS-solubilized mitochondria were submitted to the STRING database for protein–protein interaction prediction (version 11.5; https://www.string-db.org/). The parameters were set as follows: network type, “full network”; meaning of network edges, “evidence”; active interaction sources, text mining, experiments, databases, co-expression, neighborhood, gene fusion, and co-occurrence. Minimum required interaction score was set to High confidence (0.7).

**Figure 1:**
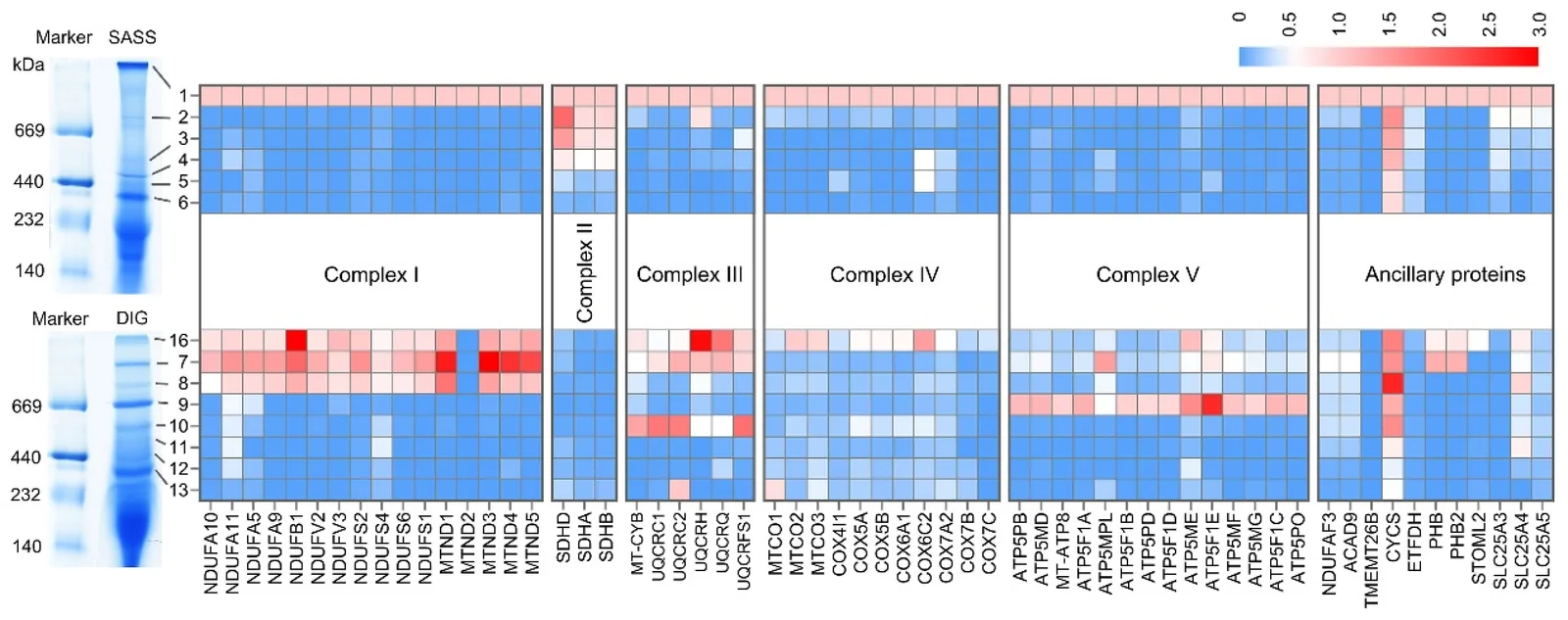
Electrophoretic distribution and relative quantitation of the OXPHOS megacomplexes subunits and ancillary proteins from mitochondria solubilized with SASS or DIG. SASS and DIG denote mitochondrial samples prepared in the presence of 4.6% SASS or 4.6% DIG, respectively.

To assess the electrophoretic distribution and relative quantitation of OXPHOS complexes on BN-PAGE gel, the relative quantitation of the subunits identified in other gel bands compared to that detected in band 1 (Figure 1) was determined using label-free quantification in Proteome Discoverer (2.5.0.400). Briefly, the relative abundance of every complex subunit detected in band 1 (Figure 1) was defined as 1. The peak areas of peptides from every complex subunit identified in other gel bands were normalized and then compared with those of the same protein in gel band 1 to determine their relative abundance ratios.

### 2.10. Purification of the OXPHOS Supercomplexes via SEC

OXPHOS supercomplexes were purified using a Waters 1525 system (Waters Corporation, Milford, MA, USA) coupled to a Waters 2489 Dual λ Absorbance Detector. A 7.5 mm × 600 mm column packed with Seplife 6FF resin (separation range: 10–4000 kDa, Sunresin New Materials, China) was used for size-based separation of supercomplexes from solubilized mitochondria. Rat liver mitochondria were resuspended in 40 volumes of ice-cold 0.05 mol/L Tris-HCl buffer (pH 7.4) containing 0.9% NaCl and 0.5 mmol/L EDTA. An aliquot of each detergent (SASS, DIG, DDM, or TX) was added, followed by vortexing and incubation at 37 °C for 10 min. The mixture was then centrifuged at 14,000 rpm for 10 min at 10 °C, and the resulting supernatants were subjected to SEC. SEC was performed using an elution buffer consisting of 0.05 mol/L Tris-HCl (pH 7.4), 0.9% NaCl, and 0.2% SASS, at a column temperature of 12 °C and a flow rate of 0.75 mL/min. The two collected chromatographic peaks were concentrated using an ultrafiltration device with a 10 kDa molecular weight cutoff and stored at −20 °C.

### 2.11. Assessment of the Integrity and Abundance of the OXPHOS Supercomplexes via Western Blot

To compare the relative percentages of OXPHOS complexes between peak 1 (P1) and peak 2 (P2) isolated from the same mitochondrial sample, two peaks eluted from a single chromatographic injection were concentrated to a final volume of 400 μL. Subsequently, equal aliquots of the P1 and P2 fractions were mixed with SDS loading buffer, heated at 99 °C for 5 min, and then loaded into adjacent wells of a 12.5% SDS-PAGE gel for electrophoretic separation. After electrophoresis, the parallel gel region containing the same complex subunit was excised and electroblotted onto a single PVDF membrane at 100 V for 40 min with an electroblotting apparatus (Junyi wet electroblotting apparatus, Junyi, China) and then immunoblotted using the corresponding specific antibody. After incubating the PVDF membrane with HRP-conjugated goat anti-mouse IgG, the target proteins were visualized using ECL detection reagent (Beyotime, CHN). The membrane was imaged using a Tanon-5200 imager with an exposure time of 0.5–3 s.

The integrated density of the Western blot (WB) bands corresponding to the same complex subunit in P1 and P2 on the same PVDF membrane was quantified using Image J software (v 1.8.0). Briefly, images were converted to an 8-bit format for uncalibrated optical density (OD) measurement. After conversion, the background subtraction was performed using the rolling ball radius method, with a consistent ball radius of 50.0 pixels applied for both samples. After image inversion, each band was individually selected and outlined, and the integrated density per unit area was measured. The integrated density of each band was then corrected by subtracting the background integrated density to obtain the final value.

The proportion of each complex subunit within the P1 and P2 fractions was determined according to the following equation:

$$\mathrm{The}\;\mathrm{proportion}\;\mathrm{of}\;\mathrm{complex}\;\mathrm{subunit}\;\mathrm{in}\;P1\;\mathrm{or}\;P2\;(\%)\;=\frac{D_{P1}\;or\;D_{P2}}{D_{P1}+D_{P2}}\times 100\%;$$

D_P1_ is integrated density of complex subunit in P1 and D_P2_ is integrated density of complex subunit in P2.

Statistical analysis was performed using repeated measures one-way analysis of variance in GraphPad Prism (10.6.0), with significance set at *p* < 0.01.

### 2.12. Co-Immunoprecipitation and Negative-Staining Electron Microscopy of Chromatographic Peak Fractions

Anti-complex I and IV immunocapture antibodies were incubated with rProtein A/G magnetic beads at 4 °C for 16 h. After removing supernatant and washing the beads, the peak P1 (isolated from the SASS-treated mitochondrial sample) was incubated with antibody-coated magnetic beads at 4 °C for 20 h. Following supernatant removal and three washes, proteins were eluted from the beads using elution buffer and subsequently analyzed by Western blot. Three biological replicates were performed according to the above procedure.

The protein solution from peak P1 (isolated from the SASS-treated mitochondrial sample) was applied to carbon-coated copper grid for 1 min and then stained by 1% of sodium phosphotungstate solution. The grid was examined using a transmission electron microscope (FEI Tecnai G2 F20, Hillsboro, OR, USA).

### 2.13. Measurement of Oxygen Consumption Rate

Measurement of oxygen consumption rate (OCR) was measured using an Agilent Seahorse XF24 extracellular flux analyzer (Santa Clara, CA, USA). To measure the OCR of the OXPHOS megacomplexes, 50 μL of P1 (≈28 μg) or 100 μL of P2 (≈118 μg), obtained from size-exclusion chromatography of SASS-solubilized mitochondria, was added into 450 μL of the assay medium (pH7.4) containing 100 mmol/L phosphate buffer, 3.5 g/L BSA, 20 mg/mL SASS, 100 μmol/L Cytochrome C and 70 μmol/L coenzyme Q10. The electron transfer reaction was initiated by adding 75 μL of 1.53 mmol/L NADH. OCR of the mitochondrial lysate was measured following the same procedure. Statistical analysis was performed using repeated measures one-way analysis of variance in GraphPad Prism (10.6.0), with significance set at *p* < 0.01.

### 2.14. Bioactive Analysis of CI, CII, and CV

CI and CII activities were assayed using a method described by Janssen et al. [37]. Briefly, 100 μL chromatographic peak P1 or P2 from SCE was pre-incubated for 3 min at 37 °C in 900 μL incubation mixture (25 mmol/L potassium phosphate, 3.5 g/L BSA, 60 μmol/L 2,6-dichloroindophenol (DCIP), 70 μmol/L decyl-ubiquinone and 20 mg/mL SASS, pH 7.8). After adding 20 μL of 10 mmol/L NADH/FADH_2_, the absorbance at 600 nm was measured every 30 s for 4 min at 37 °C using an Evolution 220 spectrophotometer (Thermo Fisher Scientific, Waltham, MA, USA). Statistical analysis was performed using repeated measures one-way analysis of variance in GraphPad Prism (10.6.0), with significance set at *p* < 0.01.

ATPase activity of CV was assayed using a method modified from Doerrler et al. [38]. Briefly, 60 μL of chromatographic peak P1 or P2 was added to 200 μL of reaction solution (25 mM Tris, pH 7.8, 150 mM NaCl, 5 mM ATP, 5 mM MgCl_2_ and 2% SASS) and incubated for 1 h at 37 °C. The reaction was stopped by adding 50 μL 12% (*w*/*v*) SDS. After centrifuging at 12,000 rpm for 5 min, 200 μL of supernatant was mixed with 60 μL of chromogenic reagent containing 0.8 mol/L sulfuric acid, 1% (*w*/*v*) ammonium molybdate and 50 mmol/L vitamin C, followed by incubation for 30 min at 37 °C. Absorbance at 620 nm was measured using a microplate absorbance reader (multiskan FC, Thermo Fisher Scientific). Three biological replicates were performed according to the above procedure. Statistical analysis was performed using repeated measures one-way analysis of variance in GraphPad Prism (10.6.0), with significance set at *p* < 0.01.

## 3. Results

### 3.1. Isolation of OXPHOS Megacomplexes from Mitochondria Enabled by SASS

**Figure 2:**
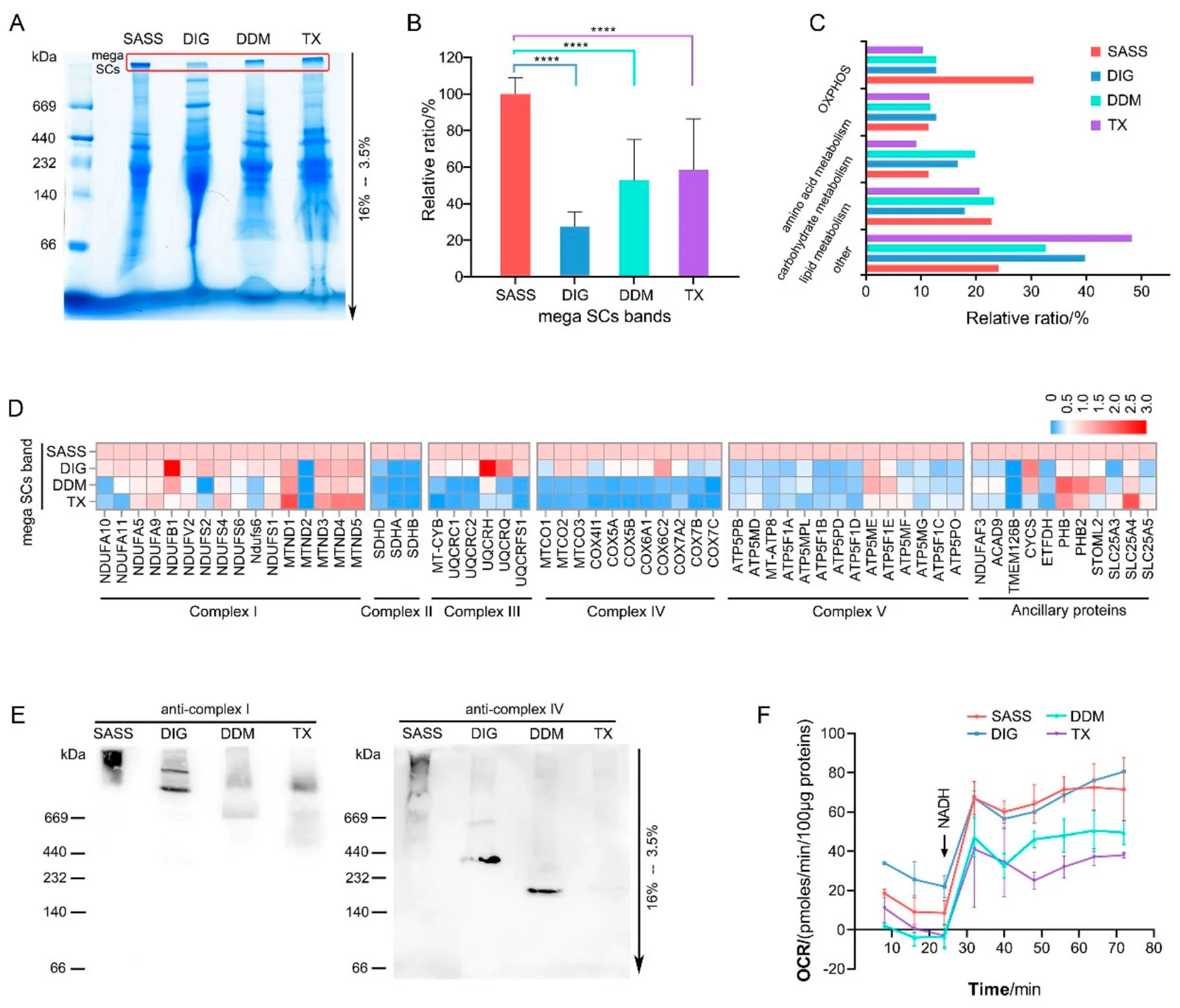
Comparative separation and identification of mitochondrial oxidative phosphorylation megacomplexes from rat liver using different detergents. SASS, DIG, DDM, and TX denote the mitochondrial samples prepared with SASS (4.6%), DIG (4.6%), DDM (2.2%), or TX (2.6%), respectively. (**A**) Representative BN-PAGE of rat liver mitochondria treated with different detergents. The mega SCs are indicated by a red frame. (**B**) Comparison of the band intensity of mega SCs from different detergents based on panel (**A**) and two additional BN-PAGE gels. ****, *p* < 0.0001 (*n* = 3 biological replicates). (**C**) Subcellular functional analysis of the highly abundant proteins in mega SCs isolated using different detergents. (**D**) Comparison of the relative quantitation of OXPHOS complex subunits and ancillary proteins among the four mega-SC bands in (**A**). (**E**) Electrophoretic distribution of two representative respiratory complexes obtained using different detergents. After BNGE, the two complexes were separately visualized by immunoblotting using sequentially specific antibodies against complexes I and IV. (**F**) OCR of the mitochondrial lysates prepared with different detergents.

To develop a novel method for isolating intact OXPHOS megacomplexes from mitochondria, we used the natural saponin SASS to disrupt rat liver mitochondria, instead of widely used DIG, DDM or TX. BN-PAGE analysis revealed abundant mega supercomplexes (mega-SCs) exhibiting the slowest relative migration rate in SASS-treated sample (Figure 2A), whereas far fewer mega-SCs were detected in lysates prepared with other detergents, particularly DIG (Figure 2B). Intriguingly, DIG-solubilized mitochondria contained multiple components exceeding 500 kDa, suggesting that the majority of mega-SCs dissociated into smaller subcomplexes during DIG-mediated solubilization (Figure 2A,B).

To characterize the composition of the mega-SCs, mega-SC bands from different mitochondrial lysates were excised individually from BN-PAGE gels, subjected to tryptic digestion, and analyzed by mass spectrometry. In mega-SC bands isolated from SASS-solubilized mitochondria, we identified approximately 80 highly abundant proteins (Sequest HT score > 99), over 30% of which were components of the five OXPHOS complexes (Figure 2C, Supplementary Data S1). By contrast, only ~12% of highly abundant proteins in mega-SC bands from other detergent-treated samples belonged to the OXPHOS system (Figure 2C, Supplementary Data S1). Notably, only SASS-isolated mega-SCs retained all five OXPHOS complexes alongside known regulatory and interacting proteins, including cytochrome c (CYCS), electron transfer flavoprotein-ubiquinone oxidoreductase (ETFDH), complex I assembly factor TMEM126B, prohibitin 1 (PHB) and prohibitin 2 (PHB2) [39], stomatin-like protein 2 (STMOL2) [40], phosphate carrier protein (SLC25A3), ADP/ATP translocase (SLC25A4), ADP/ATP translocase 2 (SLC25A5) [41,42], and several dehydrogenases involved in very long-chain acyl-CoA dehydrogenase (ACADV1) [43] and acyl-CoA dehydrogenase 9 (ACAD9) [44] (Figure 2D and Appendix A.1). In addition, available reports have confirmed that the co-migration of respiratory complexes resulted from their pre-existing physical association [14,17,18,45]. Thus, these mega-SCs, which comprise five OXPHOS complexes, are defined as OXPHOS megacomplexes. In comparison, mega-SCs isolated from DIG-solubilized mitochondria contained CI, CIII, CIV and partial CV subunits but lacked nearly all CII and most of CV (Figure 2D). Meanwhile, DDM- or TX-treated samples yielded only mega-SCs containing CI and partial CV subunits (Figure 2D, Supplementary Data S2). These conventional detergents disrupt the integrity of OXPHOS megacomplexes during mitochondrial solubilization. Collectively, these data support the hypothesis that respirasomes and ATP synthase assemble into OXPHOS megacomplex [20,21,22], and demonstrate that SASS enables the isolation of fully assembled OXPHOS megacomplexes from mitochondria.

To assess the structural integrity of detergent-extracted OXPHOS megacomplexes, we analyzed mitochondrial samples prepared with SASS, DIG, DDM, or TX using BN-PAGE followed by immunoblotting. Immunoblotting revealed that nearly all CI and CIV in SASS-treated samples co-migrated to the mega-SC band observed in Figure 2A (Figure 2E). This co-migration implied that CI and CIV assemble into OXPHOS megacomplexes. In contrast, most CI and CIV in DIG-, DDM-, or TX-solubilized samples migrated to lower molecular weight regions (Figure 2E), indicating disassembly of the OXPHOS megacomplexes. Specifically, DIG-treated mitochondria yielded CI-associated supercomplexes containing partial CIV that migrated faster than intact OXPHOS megacomplexes, whereas most CIV existed as dimers (Figure 2E)—consistent with previously reported respiratory supercomplex profiles from DIG-treated samples of mouse mitochondria [13,14,17], ovine hearts [2,23], and porcine hearts [3]. DDM- or TX-treated samples showed CI primarily as two diffuse low-molecular-weight bands, suggesting dissociation into monomers (with DDM) or severe structural disruption (with TX) (Figure 2E). Notably, mitochondrial lysates solubilized with SASS or DIG exhibited significantly higher OCR than those treated with the other two detergents (Figure 2F). Although individual respiratory complexes can catalyze redox reactions [5,9,17], our results indicate that high-order assembly is beneficial to the efficiency of redox reaction. Collectively, these data confirm that SASS preserves the integrity of OXPHOS megacomplexes, whereas conventional detergents completely disrupt these higher-order assemblies.

To compare SASS with DIG—widely regarded as the mildest detergent for supercomplex isolation [45], we analyzed high-molecular-weight (MW > 250 kDa) components from SASS- and DIG-solubilized mitochondrial lysates via mass spectrometry. SASS-treated samples yielded OXPHOS megacomplexes (band 1) containing a nearly complete complement of all five OXPHOS complexes (CI–CV) and several known ancillary proteins, with only partial dispersion of CII (Figure 1, Supplementary Data S2). This partial dissociation of CII may stem from mechanical damage to a subset of OXPHOS megacomplexes or reflect CII’s dual localization in the Krebs cycle and the respiratory chain [46]. By contrast, DIG-solubilized mitochondria yielded only smaller respiratory supercomplexes (CI–CIII–CV, CICIII, and CIII–CIV), along with free OXPHOS complexes (CI, CIII, CIV, CV) (Figure 1). Notably, no co-migration of CII with other OXPHOS complexes was observed in DIG-treated samples (Figure 1, Supplementary Data S2), confirming that DIG disrupts the integrity of OXPHOS megacomplexes during solubilization, whereas SASS preserves it.

### 3.2. Mitochondrial OXPHOS Megacomplexes Retain Structural Integrity Throughout the SASS-Based Purification Process

**Figure 3:**
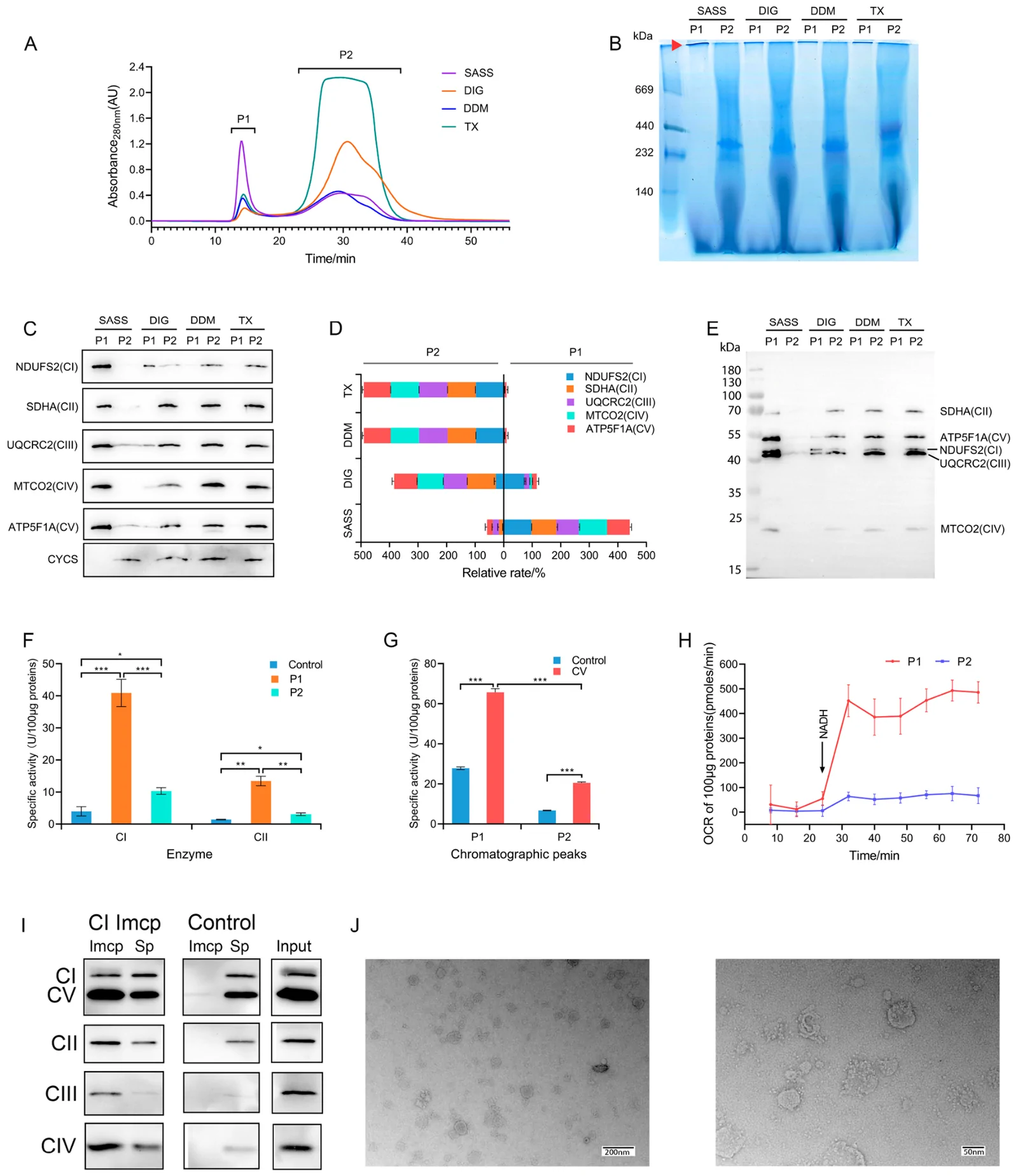
Purification and verification of mitochondrial OXPHOS megacomplexes. (**A**) Representative SEC traces of mitochondria solubilized with SASS (4.6%), DIG (4.6%), DDM (2.2%), and TX (2.6%) buffers, respectively. (**B**) BN-PAGE analysis of the chromatographic peaks collected from (**A**). OXPHOS megacomplexes are indicated by a red arrow. (**C**) Distribution of OXPHOS complexes and CYCS in chromatographic peaks P1 and P2 was sequentially detected using WB with corresponding specific antibodies (*n* = 3 biological replicates). (**D**) The relative quantity of the OXPHOS complexes and CYCS between in chromatographic peak P1 and P2 was determined based on the signal intensity of the bands in the blot shown in (**C**) (*n* = 3 biological replicates). (**E**) OXPHOS complexes in chromatographic peaks P1 and P2 were simultaneously visualized on the same PVDF membrane by WB using specific antibodies against complex I–V (*n* > 3 biological replicates). (**F**) Enzymatic activity analysis of complexes I and II in peaks P1 and P2 collected from SEC of SASS-solubilized mitochondria. The control was the reaction system without a protein sample. ***, *p* < 0.001; **, *p* < 0.01; * *p* < 0.05 (*n* = 3 biological replicates). (**G**) ATP hydrolytic activity of complexes isolated from peaks P1 and P2 collected from SEC of SASS-solubilized mitochondria. The control consisted of a protein sample added to the reaction system after the reaction had been stopped. ***, *p* < 0.001 (*n* = 3 biological replicates). (**H**) Oxygen consumption rate of equal volumes of peaks P1 and P2 collected via SEC of SASS-solubilized mitochondria (*n* = 3 biological replicates). (**I**) Co-immunoprecipitation of CI and other complexes in chromatographic peak P1 and identification of the interacting OXPHOS complexes. “Input” indicates WB analysis of complexes eluted in peak P1. “CI Imcp” indicates the immunocapture process performed in the presence of anti-complex I antibody. “Control” indicates the immunocapture process performed in the absence of anti-complex I antibody. “Imcp” and “Sp” represents the immunoprecipitation and the supernatant, respectively (*n* = 3 biological replicates). (**J**) Representative negative staining images at different magnifications of OXPHOS supercomplexes from chromatographic peak P1 of SASS-solubilized mitochondria.

Size-exclusion chromatography (SEC) was employed to purify and analyze mitochondrial OXPHOS megacomplexes isolated from rat liver mitochondria using SASS, DIG, DDM, or TX buffers. After injection of the SASS-solubilized mitochondria, numerous large supercomplexes were eluted at the void volume (peak P1) from a Seplife 6FF column (separation range: 10–4000 kDa; dimension: 7.5 × 600 mm) using Tris buffer containing 0.2% SASS. This indicates that the peak P1 contains species lager than the fractionation range of the column. The species from P1 were subsequently verified as the OXPHOS megacomplexes (consisting of CI–CV) by BN-PAGE and WB (Figure 3A–E). Quantitative analysis of representative OXPHOS subunits indicated that nearly all mitochondrial OXPHOS complexes were detected in these OXPHOS megacomplexes, accounting for 97% of CI, 80% of CII, 78% of CIII, 99% of CIV, and 79% of CV (Figure 3C–E). Indeed, co-precipitation of all five complexes was previously observed in mouse liver mitochondria solubilized with DIG using an anti-CI antibody [17]. By contrast, only trace amounts of free CII, CIII, and CV were detected in peak P2, likely originating from mechanical damage or incomplete assembly of OXPHOS megacomplexes (Figure 3C–E). Enzymatic assays further demonstrated that P1 (OXPHOS megacomplexes) exhibited significantly higher NADH dehydrogenase (CI), succinate dehydrogenase (CII), and ATP synthase (CV) activities than did the P2 fractions (Figure 3F,G). Correspondingly, P1 showed robust oxygen consumption in response to the CI substrate NADH, whereas P2 displayed minimal activity (Figure 3H). Conversely, under identical chromatographic conditions, DIG-solubilized mitochondria failed to yield intact OXPHOS megacomplexes in P1. Instead, over 99% of CII, 91% of CIV, and 80% of both CIII and CV were eluted in P2, whereas only low levels of CI, CIII, CIV, and minor amounts of CV were detected in P1 (Figure 3A–E). This aligns with previous reports using DIG to isolate similar partial respiratory supercomplexes from other species [2,3,13,14,30]. DDM- and TX-solubilized mitochondria yielded comparable profiles: nearly all OXPHOS complexes eluted in P2, with only minor amounts of CI and CV detected in peak P1 (Figure 3A–E). Notably, CYCS was absent from SEC-purified OXPHOS megacomplexes (Figure 3C), indicating negligible physical association between CYCS and these supramolecular assemblies.

To further confirm the integrity of OXPHOS megacomplexes, anti-CI immunoprecipitation was performed on SASS-derived P1 fractions. Co-precipitation of CII–CV with CI confirmed direct or indirect interactions between CI and CII, CIII, CIV, and CV (Figure 3I). In addition, the existence of respiratory supercomplexes (termed the respirasome) containing CI, CIII, and CIV has been structurally verified by several prior cryo-EM investigations [13,16,17,45,47]. Despite the absence of reciprocal immunoprecipitation, current results allow us to conclude that P1 contains fully assembled OXPHOS megacomplexes, including oxphosome. Negative-staining electron microscopy revealed that P1 of SASS-solubilized mitochondria contained quasi-spherical particles (~50–90 nm in diameter) (Figure 3J) that likely correspond to OXPHOS megacomplexes [2,3,13,45].

### 3.3. SASS Concentration and Incubation Temperature Modulate the Yield of the OXPHOS Megacomplexes

**Figure 4:**
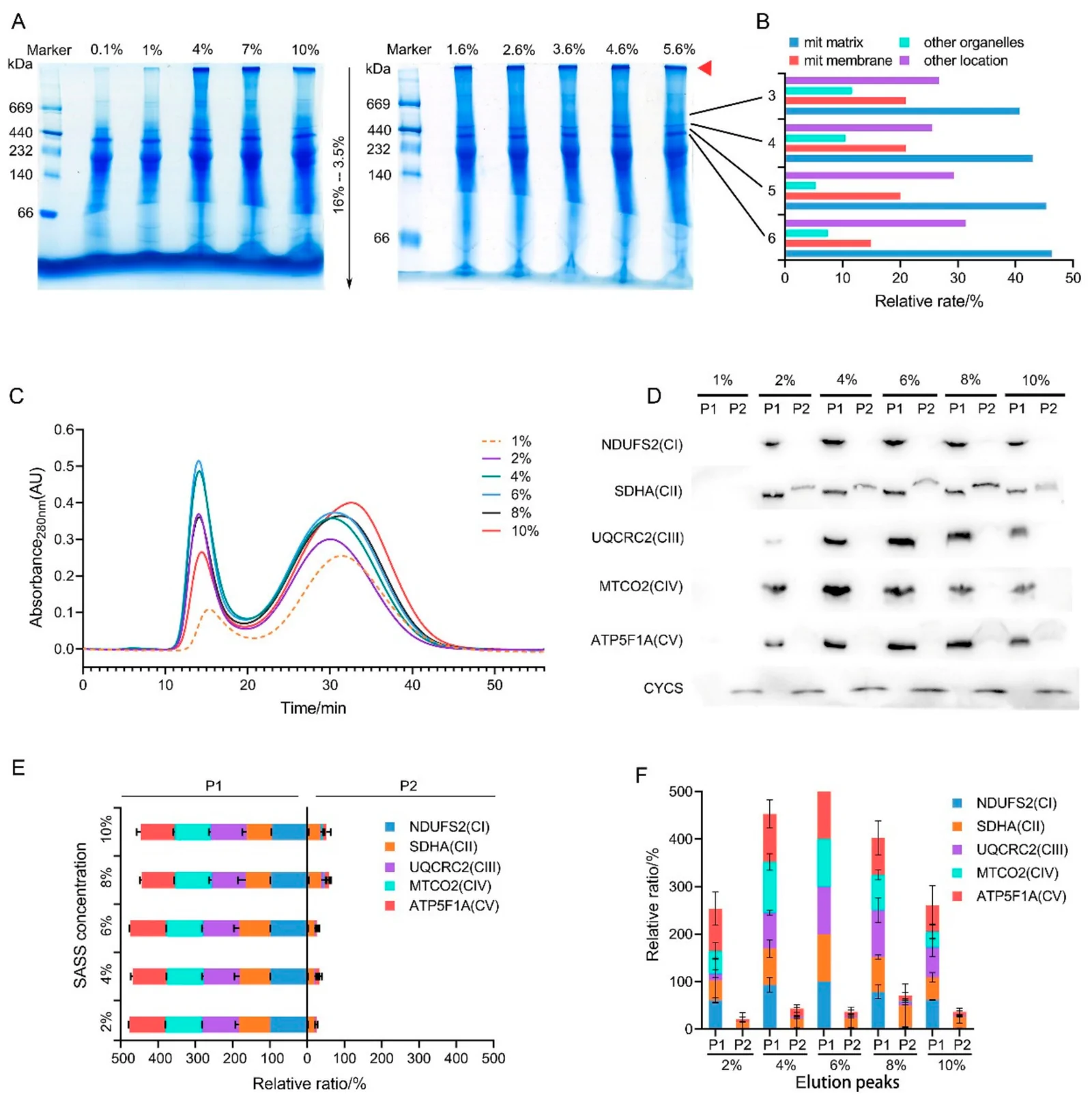
OXPHOS megacomplexes integrity and yield across a wide range of SASS concentrations. (**A**) BN-PAGE of mitochondria solubilized in 0.1% to 10% SASS (*n* > 3 biological replicates). OXPHOS megacomplexes are indicated by a red arrow. (**B**) Subcellular localization analysis of highly abundant proteins identified in bands 3 to 6. (**C**) Representative size-exclusion chromatography (SEC) traces of mitochondria solubilized at different SASS concentrations (*n* > 3 biological replicates). (**D**) WB of the chromatographic peaks P1 and P2 from mitochondria solubilized in different SASS concentrations. Specific antibodies were used to detect OXPHOS complexes and CYCS, respectively. (**E**) Relative quantities of the OXPHOS complexes and CYCS between chromatographic peaks P1 and P2 are defined based on the signal intensity of WB bands in (**D**) (*n* = 3 biological replicates). (**F**) Relative yield of OXPHOS megacomplexes harvested using different SASS concentrations.

To investigate how SASS concentration affects OXPHOS megacomplexes extraction, equal aliquots of mitochondria were treated with a gradient series of SASS concentrations, followed by analysis via BN-PAGE, SEC, and WB. The results indicated that OXPHOS megacomplexes could not be extracted from mitochondrial membranes until the SASS concentration exceeded 2% (Figure 4A,C,D), whereas the OXPHOS megacomplexes yield declined when concentration exceeded 8% (*w*/*v*) (Figure 4A,C,D). Notably, OXPHOS megacomplexes remained structurally stable across the 2–10% SASS range, with optimal integrity observed between 4% and 6% (Figure 4C–E). Collectively, these data identify 4–6% as the optimal SASS concentration range for isolating OXPHOS megacomplexes with high yield and preserved integrity. In contrast, soluble mitochondrial proteins with molecular weights of 140–500 kDa showed no concentration-dependent changes following treatment with 0.1–10% SASS (*w*/*v*) (Figure 4A). Subcellular localization analysis demonstrated that these proteins originated mainly from the mitochondrial matrix rather than the membrane (Figure 4B). Furthermore, CYCS was released from mitochondria treated with 1% SASS and did not co-purify with OXPHOS megacomplexes (Figure 4D), indicating that low SASS concentrations are sufficient to disrupt mitochondrial structure and release soluble proteins from the matrix and cristae. Finally, OXPHOS megacomplex yield and integrity were consistent across independent SASS batches, confirming the reproducibility of this extraction method (Appendix A.2).

**Figure 5:**
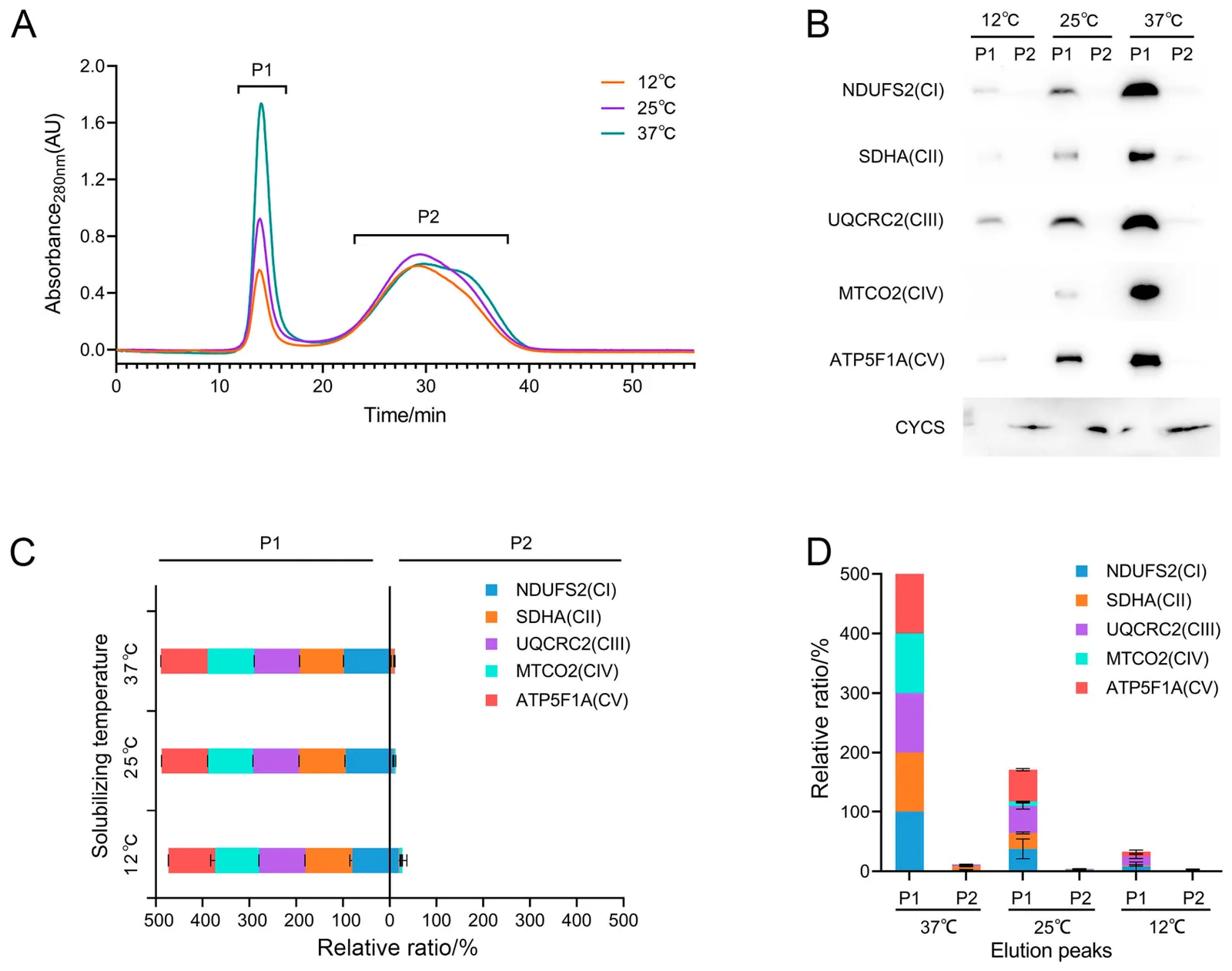
Physiological temperature improves OXPHOS megacomplexes yield without disrupting structural integrity. (**A**) Representative SEC traces of equal amounts of mitochondria solubilized in 4.6% SASS at 12 °C, 25 °C, and 37 °C, respectively (*n* > 3 biological replicates). (**B**) The distribution of the OXPHOS complexes and CYCS in chromatographic peaks P1 and P2 was detected by WB using specific antibodies (*n* = 3 biological replicates). (**C**) Relative quantities of the OXPHOS complexes and CYCS in chromatographic peaks P1 and P2 were determined based on the intensity of WB bands in (**B**) (*n* = 3 biological replicates). (**D**) Relative yield of the OXPHOS megacomplexes increasing significantly with temperature, without compromising structural integrity.

To determine the optimal incubation temperature for extracting OXPHOS megacomplexes from mitochondria, equal amounts of mitochondria were solubilized at a consistent concentration of SASS across a temperature gradient. Notably, OXPHOS megacomplexes retained structural integrity after a 10-min incubation in 4.6% SASS at temperatures ranging from 12 °C to 37 °C (Figure 5A–C), with peak yield observed at 37 °C (Figure 5A,D). Relative quantitative analysis revealed that OXPHOS megacomplexes isolated from fresh liver mitochondria solubilized in 4.6% SASS at 37 °C contained approximately 98% of CI, 94% of CII, 98% of CIII, and nearly all mitochondrial CIV and CV (Figure 5A–C). Additionally, CYCS did not co-purify with OXPHOS megacomplexes even when lysis was performed at 12 °C (Figure 5A,B), confirming the absence of a stable association between the two. Collectively, these data indicate that 37 °C is the optimal temperature for SASS-mediated mitochondrial lysis to maximize OXPHOS megacomplex yield while preserving structural integrity.

### 3.4. SASS Is Broadly Applicable to the Purification of OXPHOS Megacomplexes from Mitochondria of Diverse Origins

**Figure 6:**
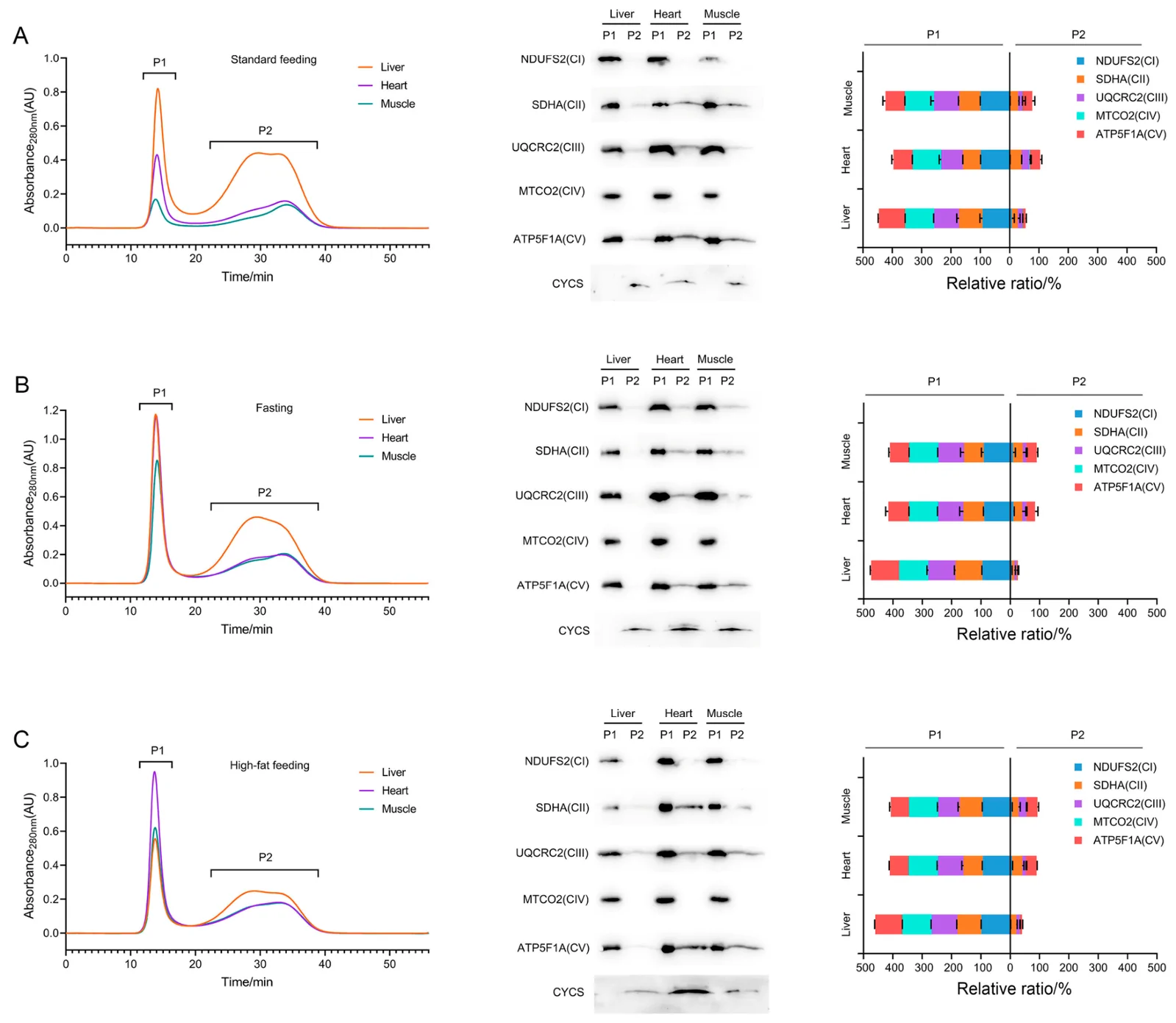
SASS enables the purification of mitochondrial OXPHOS megacomplexes from different rat tissues. Organs from six healthy rats (3 males and 3 females) of the same group were pooled prior to mitochondrial isolation to minimize individual variation (3 biological replicates). Mitochondria were solubilized by SASS lysis at 37 °C. Statistical analysis of quantified data was performed using repeated measures one-way ANOVA in GraphPad Prism 10.6.0 (*p* < 0.01). (**A**) Purification of mitochondrial OXPHOS megacomplexes from rats fed a standard diet for 24 h. Representative SEC traces of the mitochondrial lysate (**left**), the distribution of OXPHOS complexes and CYCS across chromatographic peaks P1 and P2 (**middle**), and their relative quantitation (**right**). (**B**) Purification of mitochondrial OXPHOS megacomplexes from rats fasted for 24 h (Same as above—representative SEC traces, distribution, and relative quantitation as described in panel (**A**). (**C**) Purification of mitochondrial OXPHOS megacomplexes from rats fed a high-fat diet for 24 h (Same as above—representative SEC traces, distribution, and relative quantitation as described in panel (**A**).

To assess the efficacy of SASS for isolating OXPHOS megacomplexes from diverse mitochondrial sources, mitochondria were isolated from rat organs (liver, heart, skeletal muscle) under different dietary conditions (ad libitum, 24-h fast, high-fat diet), solubilized with SASS, and analyzed via SEC and WB. For liver mitochondria, SEC-WB quantification revealed that purified OXPHOS megacomplexes contained 95% of CI, 85% of CII, 85% of CIII, 99% of CIV, and 90% of CV (Figure 6), confirming that SASS solubilization and purification preserve OXPHOS megacomplex integrity in this tissue. In contrast, heart and skeletal muscle mitochondria yielded partially damaged OXPHOS megacomplexes, with increased release of free CII, CIII, and CV. Even so, over 70% of CII, 80% of CIII, 65% of CV, and nearly all CI and CIV remained associated with the OXPHOS megacomplexes (Figure 6). This partial damage likely results from the extensive tissue grinding required to isolate mitochondria from heart and skeletal muscle, which may compromise the mitochondria prior to SASS treatment. Consistent with prior reports [48], CII, CIII, and CV are also inherently more prone to dissociate from OXPHOS megacomplexes under mechanical stress. Additionally, higher levels of CII, CIII, and CV in muscle and heart may reflect tissue-specific differences. Notably, OXPHOS megacomplex abundance and integrity were unaffected by 24-h fasting or a high-fat diet across all tissues. Additionally, CYCS was not detected in any purified OXPHOS megacomplex fractions (Figure 6), further confirming the specificity of SASS-mediated isolation. Collectively, these data demonstrate that SASS is a reliable tool for isolating largely intact OXPHOS megacomplexes from mitochondria across multiple rat tissues, even from samples subjected to mechanical processing or dietary perturbations.

## 4. Discussion

Mitochondria are the primary sites of cellular energy production and possess a highly folded inner membrane architecture known as cristae, which houses the OXPHOS system responsible for bioenergetics. Many studies have attempted to elucidate the assembly models of this system [1,2,3,13,17,42]; however, the actual architecture of the mitochondrial OXPHOS system remains uncertain because of challenges in determining how individual complexes assemble [1]. Although interactions between ATP synthase and certain respiratory complexes or supercomplexes have been observed in rat heart and human fibroblast mitochondria using immunoprecipitation or cryo-electron tomography [17,22,49], attempts to isolate a physical entity encompassing both respiratory complexes and ATP synthase have consistently failed [1]. The major hurdle is the inability to preserve supercomplex integrity during solubilization and separation from mitochondrial membranes.

Solubilization of OXPHOS supercomplexes under strongly hydrophobic conditions typically requires detergents [17,50], yet the composition of the resulting supramolecular assemblies varies considerably with the detergent employed [17]. Earlier studies have reported detergent-induced fragmentation of supercomplexes [17]. Currently, DIG is widely regarded as the mildest detergent for extracting respiratory supercomplexes and ATP synthase from mitochondria [2,3,13,14,51]. Our observations using DIG, DDM, and TX buffers to extract mitochondrial supercomplexes are consistent with earlier reports: DDM and TX buffers tend to yield free complexes [2,10,13,17,23,28,30,52], whereas DIG extracts both respiratory supercomplexes and free complexes [1,4,10,12,14,17,32,52,53,54,55,56,57]. In contrast, when SASS was used to lyse the same mitochondria, the OXPHOS megacomplexes separated and purified from the lysates comprised nearly all OXPHOS complexes and displayed both respiratory and ATP hydrolysis activities. Co-migration of these OXPHOS megacomplexes with known auxiliary proteins of respiratory complexes and ATP synthase (e.g., prohibitin [39], phosphate carrier, and ADP/ATP carrier [41,42]) further indicates that SASS effectively preserves protein–protein interaction throughout the separation and purification process. Some OXPHOS subunits were not identified in the mega-SC band (Figure 2A), likely because of their low abundance or loss during solubilization. Importantly, these megacomplexes are likely large protein particles, as they can traverse the 3% stacking gel and enter the 3.5% separating gel, albeit with limited mobility because of their large size [23,45,58]. By contrast, large cristae membrane fragments containing numerous respirasomes, ATP synthases, and other membrane proteins would be expected to remain trapped in the well or stacking gel. Importantly, these megacomplexes remain stable even when mitochondrial lysates are incubated at 37 °C for 10 min. Of course, the possibility that they arise from extraction-induced assembly cannot be ruled out until further experimental evidence confirms otherwise. The absence of physical association between CYCS and OXPHOS megacomplexes suggests that SASS-mediated solubilization can disrupt weak or transient interactions, possibly also leading to the loss of OXPHOS megacomplex components and alteration of megacomplex integrity. Additionally, mechanical damage incurred during mitochondrial isolation may contribute to megacomplex disruption. Homogenization is essential for isolating mitochondria from vertebrates and yeast; however, the I–II–III_2_–IV_2_ supercomplex has never been observed in these organisms, even with DIG extraction [2,3,53]. In contrast, this supercomplex was isolated from *Tetrahymena thermophila* mitochondria, which do not require homogenization during isolation [45]. Consistent with this notion, our results revealed higher levels of free CII, CIII, and CV in mitochondrial lysates from muscle and heart tissues than from liver (Figure 6). Collectively, our data demonstrate that the SASS-based separation technique offers a unique advantage for extracting OXPHOS megacomplexes from mitochondria. Furthermore, this method holds promise for the universal isolation of protein megacomplexes from diverse biomembranes, potentially enabling the identification of novel supramolecular architectures for future structural biology investigations.

In our study, co-precipitation of CII–CV with CI confirmed a physical association between CI and CII, CIII, CIV, and CV (Figure 3I). Notably, the CI–CV complexes precipitated by the anti-CI antibody were derived from OXPHOS megacomplexes purified by SEC. In addition, the existence of respiratory supercomplexes (termed the respirasome) containing CI, CIII, and CIV has been structurally verified by several prior cryo-EM investigations [2,13,16,18,45,47,59]. Despite the absence of reciprocal immunoprecipitation, the current results allow us to conclude that peak P1 contains fully assembled OXPHOS megacomplexes, including oxphosome. This suggests that oxphosomes may be composed of a respirasome (CICIII_2_CIV_2–4_, ~2MDa) [13,28], dimeric CII [28] and ATP synthase with a phosphate carrier (PIC), and an adenine nucleotide carrier (CV_2–6_, ~2MDa) [42,51]. Oxphosomes are effectively solubilized and preserved by SASS during mitochondrial lysis, whereas they disassemble in DIG, DDM, or TX buffers. Previously, the CI–CII–CIII_2_–CIV_2_ supercomplex, with a MW of approximately 2 MDa, was considered the largest respiratory supercomplex and was purified from DIG-solubilized *T. thermophila* mitochondria [45]. However, even with DIG solubilization, most CII and CV are typically found in their free forms [14,17]. The existence of oxphosomes is further supported by functional evidence [31] and the in situ arrangement of the respirasome and ATP synthase [22]. Of course, whether oxphosomes represent pre-existing native structures or arise from SASS-induced artifacts during sample preparation remains to be further verified.

A long-standing question in membrane biology concerns the distribution, abundance, and physiological variation of OXPHOS supercomplexes in response to disease, environmental conditions, and aging [12,60,61,62,63]. Earlier reports indicated that approximately 80% of CI, 65% of CIII, and 15% of CIV assemble into respiratory supercomplexes, whereas most CII and CV remain as free complexes [10,57]. These findings were previously interpreted as supporting the plasticity model over the solid-state and fluid-state models [1,5]. However, our results are not fully consistent with either the plasticity model or fluid-state model: in liver mitochondria, nearly all OXPHOS complexes are detected within OXPHOS megacomplexes isolated via SASS. Although a small fraction of free CII, CIII, and CV was detected in SASS-solubilized heart and muscle mitochondria, most OXPHOS megacomplexes retained their structural integrity. Notably, these megacomplexes associate with specific chaperone proteins that likely contribute to their stability and efficient oxidative phosphorylation (Figure 2) [39,40,41,42,43,44]. Most importantly, these megacomplexes remained stable following 24 h of fasting or high-fat diet exposure. Of course, because the combined processing of the same organs from six animals would obscure individual differences, further studies are needed. Collectively, our data demonstrate that SASS preserves OXPHOS megacomplex assemblies during extraction, suggesting that these assemblies represent the predominant organization in SASS-solubilized mitochondria. However, it remains uncertain whether these megacomplexes represent pre-existing native architecture or are assemblies induced by SASS, as in situ structural evidence is currently lacking.

## 5. Conclusions

This study introduces a novel isolation technique for membrane protein supercomplexes, enabling the efficient purification of OXPHOS megacomplexes from mitochondria. Because these megacomplexes incorporate nearly all respiratory complexes and ATP synthases within mitochondria, they likely represent the predominant structural organization of the OXPHOS system. These findings provide critical new insights into OXPHOS architecture, suggesting that the system operates as a coordinated network of OXPHOS megacomplexes that collectively retain robust complex I, complex II, and ATP synthase enzymatic activities. Moreover, the successful isolation of oxphosomes provides a foundation for future studies exploring the molecular architecture and mechanistic basis of the OXPHOS system. Collectively, the data support isolation of high-molecular-weight assemblies containing complexes I–V, while their precise native organization and physiological significance remain to be established. Given its ability to preserve supercomplex integrity, this technique also holds promise for structural and functional analyses of other membrane protein supercomplexes across diverse biological systems.

## Figures and Tables

**Figure A1:**
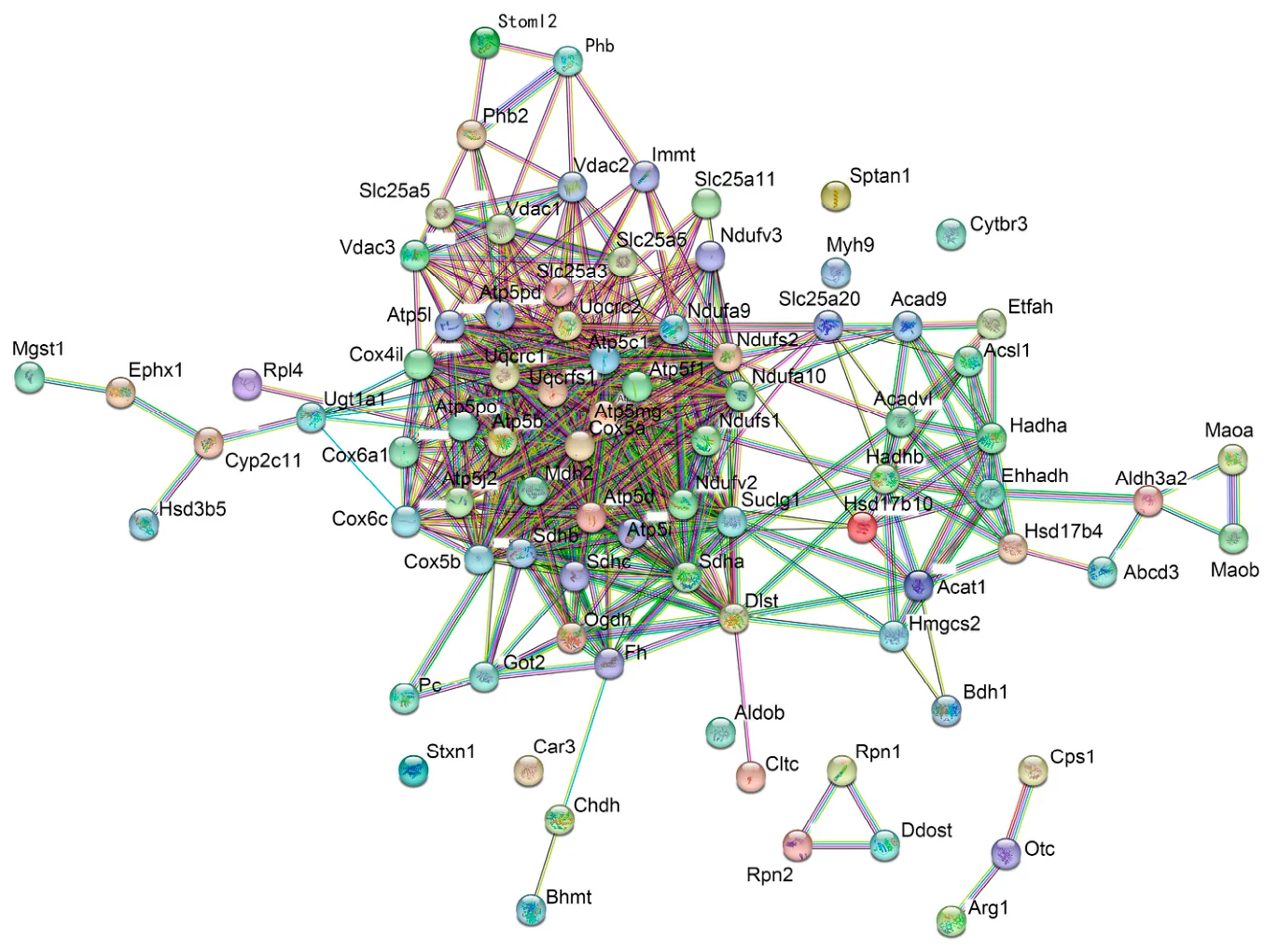
Interaction prediction of highly abundant proteins identified from mega-SC band of SASS-solubilized mitochondria. The accession numbers of identified proteins from the mega-SC band were submitted to the STRING database (version 11.5; https://www.string-db.org/). The following parameters were used: Network type: full network; Meaning of network edges: evidence; Active interaction sources: Textmining, Experiments, Databases, Co-expression, Neighborhood, Gene Fusion, and Co-occurrence. Minimum required interaction score: high confidence (0.7).

**Figure A2:**
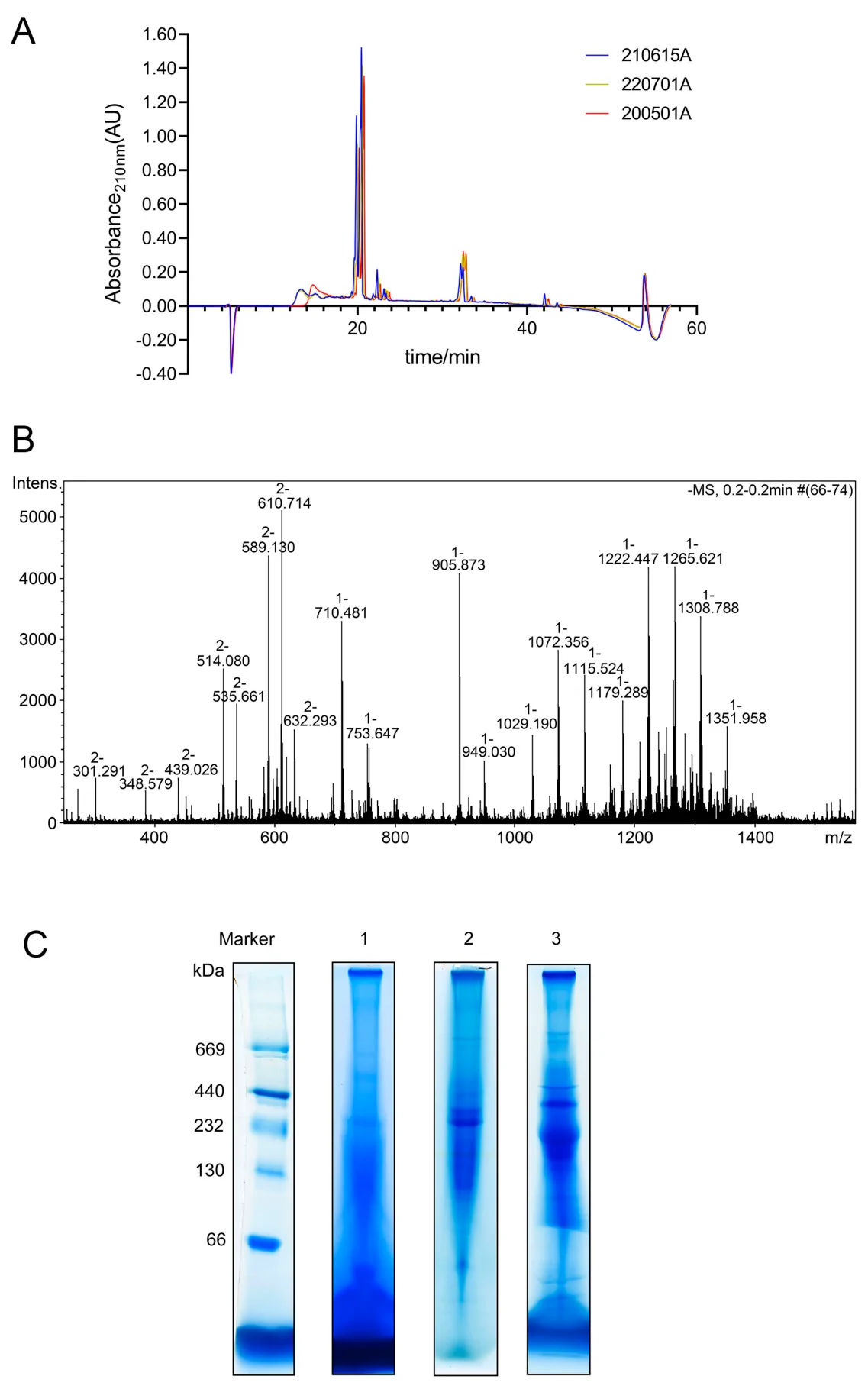
Reproducible production of SASS ensures reliable separation of the OXPHOS megacomplexes from mitochondria. (**A**) Representative HPLC traces of different of SASS lots. For reverse-phase HPLC (RP-HPLC), eluant A was water with 0.1% trifluoroacetic acid (TFA), and eluant B was acetonitrile with 0.1% TFA. Components were separated over 57 min using the following gradient of eluant B: 0–13 min, 35%; 13–41 min, 35–70%; 41–46 min, 70–90%; 46–47 min, 90–0%; 47–57 min, 0%. (**B**) Representative mass spectrum of SASS. The mass spectrum was acquired in negative-ion mode using a micrOTOFQ-II mass spectrometer (Bruker, Billerica, MA, USA). (**C**) BN-PAGE of mitochondria solubilized with different lots of SASS. OXPHOS supercomplexes were indicated by red arrow. SASS lots used: 1, lot No. 210615A; 2, lot No. 200501A; 3, lot No. 220701A.

## Data Availability

The raw data and search results of mass spectrum (project ID: IPX0013464000) can be acquired from iProX at https://www.iprox.cn/page/project.html?id=IPX0013464000 (accessed on 12 September 2026).

## Supplementary Materials

The following supporting information can be downloaded at: https://www.mdpi.com/article/10.3390/biology15201826/s1. Supplementary Data S1. Mass spectrometry search results for bands excised from BN-PAGE gels. The files Duan-1-20210110 through Duan-20-20210110 contain the mass spectrometry search results corresponding to bands 1–20 in Figure 1. Supplementary Data S2. Relative abundance ratios of the same protein in other bands versus that in band 1 of Figure 1.

## Abbreviations

The following abbreviations are used in this manuscript:

OXPHOS	oxidative phosphorylation
SASS	*Sapindus saponaria* saponins
CI–CIV	complex I–complex V
DIG	Digitonin
DDM	dodecylmaltoside
TX	Triton X-100
CYCS	cytochrome c
