# Supplementary material for: Saponin-Mediated Isolation of Megacomplexes of the Mitochondrial Oxidative Phosphorylation System
Source: Biology (Basel). 2026 Oct 13;15(20):1826. doi: 10.3390/biology15201826 (PMC13701924; doi:10.3390/biology15201826)
Supplement: Supplementary file 1 [file biology-15-01826-s001.zip › biology-4592065-WB/Figure 3I/introductions for Co-IP images.pdf]

The first row shows immunoblot bands probed with anti-CI antibody, while the second row displays those probed with anti-CV antibody. Immunoprecipitates and supernatants of experiment group are loaded in the first and second columns, respectively, with 3 biological replicates arranged from columns 1 to 6. The 7th and 8th columns contain the immunoprecipitate and supernatant of the control group.
